# Occurrence of Emerging Micropollutants in Water Systems in Gauteng, Mpumalanga, and North West Provinces, South Africa

**DOI:** 10.3390/ijerph14010079

**Published:** 2017-01-13

**Authors:** Elijah M. M. Wanda, Hlengilizwe Nyoni, Bhekie B. Mamba, Titus A. M. Msagati

**Affiliations:** Nanotechnology and Water Sustainability Research Unit, College of Science Engineering and Technology, University of South Africa, P.O. Box 392, UNISA 003, Florida, Roodepoort 1709, Johannesburg, South Africa; elijahwanda@gmail.com (E.M.M.W.); nyonih@unisa.ac.za (H.N.); Mambabb@unisa.ac.za (B.B.M.)

**Keywords:** drinking water, emerging micropollutants, GCxGC-HRTOFMS, spatial variations, wastewater

## Abstract

The ubiquitous occurrence of emerging micropollutants (EMPs) in water is an issue of growing environmental-health concern worldwide. However, there remains a paucity of data regarding their levels and occurrence in water. This study determined the occurrence of EMPs namely: carbamazepine (CBZ), galaxolide (HHCB), caffeine (CAF), tonalide (AHTN), 4-nonylphenol (NP), and bisphenol A (BPA) in water from Gauteng, Mpumalanga, and North West provinces, South Africa using comprehensive two-dimensional gas chromatography coupled to high resolution time-of-flight mass spectrometry (GCxGC-HRTOFMS). Kruskal-Wallis test and ANOVA were performed to determine temporal variations in occurrence of the EMPs. Principal component analysis (PCA) and Surfer Golden Graphics software for surface mapping were used to determine spatial variations in levels and occurrence of the EMPs. The mean levels ranged from 11.22 ± 18.8 ng/L for CAF to 158.49 ± 662 ng/L for HHCB. There was no evidence of statistically significant temporal variations in occurrence of EMPs in water. Nevertheless, their levels and occurrence vary spatially and are a function of two principal components (PCs, PC1 and PC2) which controlled 89.99% of the variance. BPA was the most widely distributed EMP, which was present in 62% of the water samples. The detected EMPs pose ecotoxicological risks in water samples, especially those from Mpumalanga province.

## 1. Introduction

Emerging micropollutants (EMPs) are ubiquitous in aquatic environments and are a matter of growing concern worldwide [1,2,3,4,5,6,7,8,9,10]. The EMPs comprise of a wide range of natural and synthetic organic compounds, which include pharmaceuticals and personal care products (PPCPs), detergents, steroid hormones, industrial chemicals, pesticides, and many other contaminants of emerging concern [2,3,5,7,9]. Compared to other contaminants of anthropogenic origin, the EMPs have largely been outside the scope of monitoring and regulation worldwide, and there is paucity of data on their levels, occurrence, and fate in water [1,2,4,6,8,9,10,11,12,13,14,15,16,17,18,19,20]. Nevertheless, the ubiquity occurrence of EMPs has been increasingly identified in both surface and ground water sources as a result of influx of effluents from wastewater treatment plants (WWTPs), on-site wastewater disposal systems, runoff from roadways wash off, agriculture fields, recreational activities, atmospheric deposition, animal feeding operations, leaking sewer lines, and landfill and septic tank leachate [8,10,21,22,23,24,25,26,27,28,29].

The majority of the existing WWTPs are not particularly constructed with the intention to remove EMPs from wastewater streams [10,30,31,32]. Consequently, the EMPs pass through WWTPs owing to their hydrophilicity, partial degradation, persistency, and the continuous introduction into the drinking water value chains of the hydrological cycle coupled with lack of well-established precautionary measures and monitoring actions [10,30,31,32]. Once the EMPs find their way into ground and surface water environments, they may re-enter the biosphere where they pose a threat to plant and animal life in addition to posing challenges to the potable water treatment facilities [10,33]. Despite their occurrence at low concentrations (i.e., from pg/L to μg/L) in water environments, the EMPs are associated with several ecotoxicological effects, such as chemo-sensitization, endocrine disruption, disruption of the production of platelets, red and white blood cells, short and long term toxicity, antibiotic resistance of microorganisms, and insomnia among others [1,3,4,6,8,9,10,11,12,13,14,15,16,17,18,19]. Severe ecotoxicological effects of EMPs have been reported to be as a result of the interactions of EMPs with human and/or aquatic life usually after long-term exposure to the EMPs [13,15,34]. Among these EMPs, Bisphenol A (BPA) (C_15_H_16_O), 4-Nonylphenol (NP) (C_15_H_24_O), carbamazepine (CBZ) (C_15_H_12_N_2_O), caffeine (CAF) (C_8_H_10_N_4_O_2_), galaxolide (HHCB) (C_18_H_26_O), and tonalide (AHTN) (C_18_H_26_O), present significant research interests due to their extensive use in several products, their ecotoxicological effects, as well as their physicochemical properties which allow their persistent presence in water [7,8,11,13,15,16] (Table 1). While BPA and NP are endocrine disrupting compounds, HHCB and AHTN are chemo-sensitizers (they have the potential to interfere with transportation of proteins (P-glycoprotein) and inhibition of cellular defence mechanisms), CBZ has the potential to disrupt the production of red blood cells, white blood cells, and platelets, and CAF has the potential to disrupt sleep (insomnia) (Table 1).

According to Musolff et al. [8], an understanding of spatial and temporal variations in concentration heterogeneities of EMPs is one of the most important aspects in the study of EMPs. Nevertheless, the levels and occurrence of EMPs in the environment are largely ill understood in many parts of the world [20]. Generally, surface waters and wastewaters have been assumed to contain diverse and higher levels of EMPs compared to groundwater [20]. Lapwrorth et al. [20] noted that this assumption and the resulting paucity of data on occurrence of EMPs in water might simply be a function of limitations in capacity of several analytical methods and equipment, as well as the limited number of research studies rather than actual levels and occurrence of EMPs in water. With the advent of novel and highly sensitive analytical equipment capable of detecting chemical constituents at very low levels in complex sample matrices, the detection of EMPs is now possible, and has thus attracted the attention of researchers worldwide [1,3,4,6,8,9,10]. Traditionally, mass spectrometry (MS) or selective detectors coupled with gas or liquid chromatography had to be used for the identification of EMPs. However, one of the problems associated with these analytical techniques in the identification of these organic compounds, including EMPs, has been the co-elution of several compounds and their inability to quantify the compounds below traditionally reported limits of detection [35]. Another limitation of these traditional techniques is the additional time required and material needed for analysis when repeating the analysis on another column with different polarity [35].

The LECO Pegasus^®^ 4D comprehensive two-dimensional gas chromatography coupled to high resolution time-of-flight mass spectrometry (GC × GC-HRTOFMS) is a recently developed robust state-of-the-art analytical technique that offers a solution to the co-elution problem with minimal time and material requirements for analysis [36]. The GC × GC-HRTOFMS (two-dimensional gas chromatography coupled to high resolution time-of-flight mass spectrometry) has proven to be one of the most powerful analytical techniques for the analysis of environmental samples and plays a significant role in the determination of organic compounds, including EMPs, in environmental samples [36,37,38,39]. According to LECO Corporation (St. Joseph, MI, USA) [36], the GC × GC-HRTOFMS system utilises four dimensions of separation and resolution, namely: (1) chromatographic resolution in first dimension; (2) chromatographic resolution in second dimension; (3) exceptional mass accuracy of 1 ppm as well as amplified mass resolution; and (4) high resolution deconvolution^®^ (HRD^®^) available in the ChromaTOF-HRT^®^ software package (LECO Corporation, St. Joseph, MI, USA) which is used to operate the GC × GC-HRTOFMS system. The ChromaTOF-HRT^®^ software package coupled with increased peak capacity of the GC × GC at least two times greater than any other product on the market allows environmental samples to be handled in such a way that compound identification and quantification are not compromised. In addition, the GC × GC-HRTOFMS produces data with much improved separation capacity, signal-to-noise (S/N) ratio, chemical selectivity, and sensitivity [36,37,39,40,41,42].

In principle, the method consists of two GC systems equipped with columns of different polarity connected by an interface with an integrated cryogenic trap [35,36]. The cryogenic trap repeatedly condenses compounds eluting from the primary column and releases them periodically as short pulses to the secondary column [35]. Parameters like duration and frequency of both condensation and injection pulses are variable and allow precise tuning of the instrument according to the requirements of the analysis. Since GC × GC produces very narrow peaks (down to 50 ms, depending on the frequency of cryogenic modulation) a HRTOFMS detector capable of mass resolutions of up to 50,000 with a high acquisition rate (up to 200 spectra/s) is utilised [36]. The HRTOFMS system utilises a chemical ionisation source (HR-CI) which further enhances the system accuracy as well as high resolution on pseudo-molecular ions, which substantiates the conventional electron ionisation source (HR-EI) which provides the comprehensive characterisation of unknown compounds [36].

Despite the enhanced detectability and reliability of the GC × GC-HRTOFMS in the identification and quantification of EMPs in complex environmental samples owing to the two retention times and well-ordered bands of compound groups in the GC × GC system [36,43], there has been very little, if any, focus on the monitoring and determination of EMPs in groundwater and surface water sources used by thousands of people for their domestic needs in Gauteng, Mpumalanga, and North West provinces. However, these water sources are also at risk of contamination by a variety of contaminants including EMPs. As of yet, no lasting solutions have been proposed to address the problems associated with EMP contamination in many parts of the world, including Gauteng, Mpumalanga, and North West provinces in South Africa.

Due to the aforementioned widespread use, occurrence, distribution, fate, and effects of EMPs, sensitive and selective multi-residue analytical methods and techniques are required that will allow detection in environmental samples. The objectives of this study were therefore to: (1) determine the levels and occurrence of the analytes (i.e., BPA, CAF, CBZ, HHCB, NP, and AHTN) in the solid phase extraction (SPE) extracts using comprehensive GC × GC-HRTOFMS in water samples from Gauteng, Mpumalanga, and North West provinces, South Africa; (2) determine the limit of detection (LOD) and limit of quantification (LOQ) for BPA, NP, CAF, HHCB, AHTN, and CBZ by using comprehensive GC × GC-HRTOFMS; and (3) determine the temporal and spatial variations in the occurrence of the analytes during the study period.

## 2. Materials and Methods

### 2.1. Materials and Preparation of Reagents

The chemicals, BPA standard, NP standard, CAF standard, HHCB standard, AHTN standard, CBZ standard, methanol LC-MS CHROMASOLV^®^, and dichloromethane LC-MS CHROMASOLV^®^, hydrochloric acid (HCl), sodium hydroxide (NaOH), and standard pH buffers (for pH 4 and 7) were obtained from Sigma-Aldrich, Johannesburg, South Africa. All chemicals were used without further purification.

### 2.2. Study Area and Water Sample Collection

#### 2.2.1. Study Area

**Figure 1:**
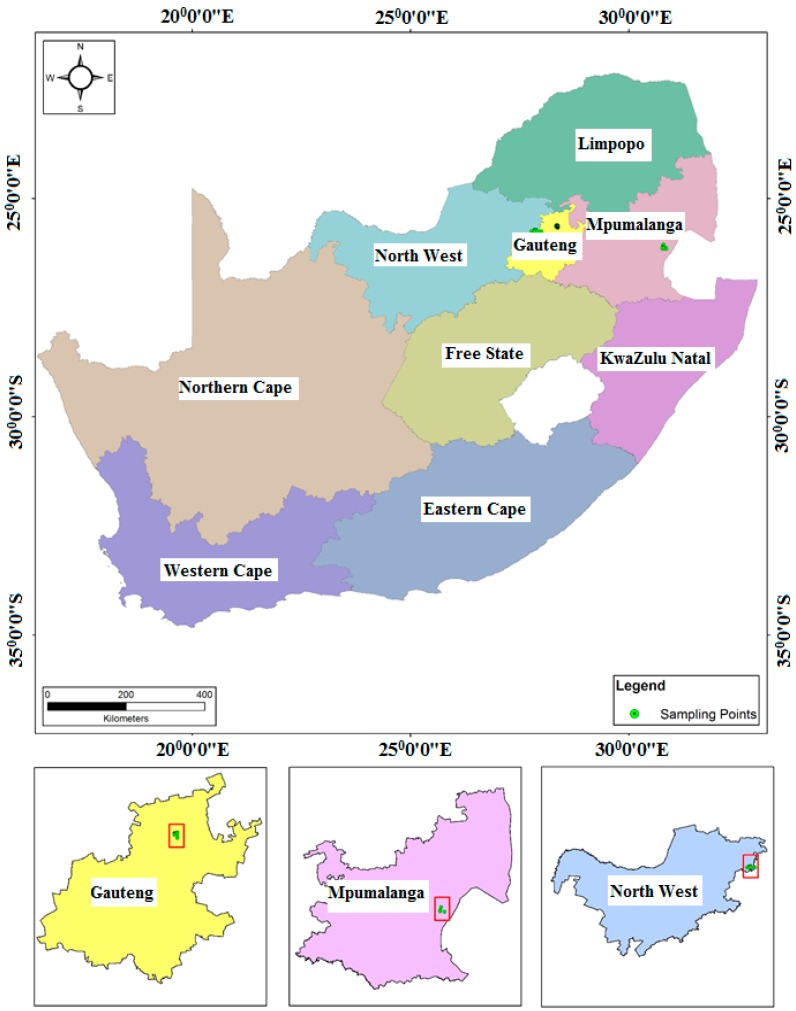
Map of South Africa showing location of Mpumalanga, Gauteng, and North West provinces and sampling points.

The areas selected for study were Mpumalanga, Gauteng, and North West provinces in South Africa (Figure 1). Gauteng is the smallest province in South Africa, which covers an area of 18,178 square kilometres. Gauteng province is bordered by the North West, Limpopo, Free State, and Mpumalanga provinces. It is the most populated province with a total population of 12,272,263 [44]. Mpumalanga province is located to the eastern part of South Africa and is surrounded by Swaziland and Mozambique on the eastern side, and Gauteng province on the western side (Figure 1). Mpumalanga province covers an area of 79,487 square kilometres [44]. Mpumalanga province has a total population of 4,039,939 [44]. The North West province is located at the central part of South Africa and bordered by the Northern Cape on the southwestern side, the Free State to the southern part, Gauteng to the eastern side, and Limpopo to the northeastern side, with Botswana on its northern border (Figure 1). North West province has a total surface area of 116,231 square kilometres [44]. North West has a total population of 3,509,953 [44]. The increase in population in all the three provinces has resulted in increased water consumption, which aggravates pressure on South Africa’s existing water resources. In addition, Mpumalanga and North West provinces are faced with acute water resource constraints because they are largely arid provinces. Furthermore, based on both the Blue Drop Report of 2012 and the Green Drop Report of 2012, the majority of water supply sources in North West and Mpumalanga provinces were not fit for human consumption [45,46].

#### 2.2.2. Water Sample Collection and Onsite Water Sample Analyses

In this study, water samples were collected once every two months between June 2014 and April 2016. A total of 44 quasi-randomly selected locations within drinking water and wastewater sources in Mpumalanga, Gauteng, and North West provinces were utilized. Sampling sites were selected taking into account the variations in physiography and anthropogenic activities around the selected sites in each of the three provinces. In Mpumalanga province, groundwater and surface water samples were collected from school boreholes, shallow wells, the Eerstehoek water treatment plant (WTP), effluent from the Eerstehoek wastewater treatment plant (WWTP), and rivers located in the low-income areas situated in the Chief Albert Luthuli municipality in Mpumalanga, close to the Oshoek border between Swaziland and South Africa (Figure 1). In Gauteng and North West provinces, water samples were collected from rivers, Roodeplat and Hartbeespoort dams, and the Schoemansville water treatment plant. For analysis of EMPs (BPA, NP, CAF, HHCB, AHTN, and CBZ) the sample bottles were rinsed twice with water from a particular sampling site before obtaining the final sample. Grab water samples were collected using 1 L glass bottles in triplicate using standard sampling procedures. The water samples were analyzed for levels of pH, electrical, conductivity (EC), and total dissolved solids (TDS) in the field immediately after sampling, using a Hanna Instrument (Woonsocket, RI, USA) model HI-9828 multi-meter. Deionized water was used to rinse the electrode of the meter prior to determination of the levels of TDS, EC, temperature, and pH of any successive sample to avoid inter-sample contamination.

### 2.3. Preparation of Standard Solutions, Sample Preparation, and Solid Phase Extraction of Emerging Micropollutants

#### 2.3.1. Preparation of Standard Solutions

The 1000 mg/L stock solutions for BPA, NP, HHCB, AHTN, and CBZ were prepared by weighing 1 mg of each into a vial (1.5 mL) and dissolving the sample in methanol (LC-MS CHROMASOLV^®^ grade) (1 mL) under vortex and ultrasonication for 10 min. In addition, 1000 mg/L stock solutions for CAF were prepared by weighing 1 mg of CAF into 1.5 mL vial followed by dissolving the sample in dichloromethane (LC-MS CHROMASOLV^®^ grade) (1 mL) under vortex and ultrasonication for 10 min. The stock solutions were stored in a refrigerator at a temperature of below 4 °C. From each of the stock solutions, 100 µL was pipetted and placed into one 1.5 mL vial and made up to the 1.0 mL mark with methanol (LC-MS CHROMASOLV^®^ grade) to prepare a 100 mg/L mixed standard solution from which all working standards of different concentrations (10, 20, 30, 40, and 50 parts per million (ppm)) were prepared for the preparation of the calibration curve.

#### 2.3.2. Sample Preparation and Solid Phase Extraction of Emerging Micropollutants

The water samples were kept in cooler boxes under ice and transported to the laboratory where they were filtered using a 1.2 μm GF/C Whatman filter paper to remove suspended matter prior to autotrace solid phase extraction (SPE) treatment and stored in a refrigerator below 4 °C until analysis. Solid phase extraction was used to extract, clean, and enrich/pre-concentrate the analytes in water samples using Dionex autotrace 280 by Thermo Scientific. The autotrace-SPE system was optimized with regard to initial analyte dose, sample pH, and sample volume. The EmporeTM Styrene Divinyl Benzene (SDB-RPS) autotrace-SPE disks were conditioned with methanol and deionised water. One hundred millilitres of water samples containing the target analytes were loaded onto the autotrace SPE and eluted through the SPE disks followed by washing with 5 mL of deionised water. The SPE disks were then subjected to vacuum drying in order to remove excess water before eluting the compounds with methanol (5 mL). The extracted solution was evaporated to dryness using nitrogen gas before reconstituting with methanol (1 mL) for GC × GC-HRTOFMS analysis [47].

The SPE system was optimized with regard to initial analyte dose, sample pH, and sample volume. The initial analyte dose of 5 µg/L, 10 µg/L, and 15 µg/L for each analyte into actual water samples as well as ultra-pure water samples was investigated via the standard addition method while keeping sample pH, sample volume, volume of methanol, and flow rate constant. The sample pH of the actual water samples and ultra-pure water was measured using Hanna model HI-9812 multi-meter (Hanna Instruments Limited, Bedfordshire, UK). The electrode of the meter was rinsed with deionised water before determining pH of any subsequent sample to prevent inter-sample contamination. Sample pH was optimized by varying the pH of actual water samples and ultra-pure water spiked with 1000 μg/L of each analyte. The pH was adjusted to 2, 4, 5, 7, 8, and 10 with HCl (1 mol/L) and NaOH (0.6 mol/L) [48].

The effect of sample volume was investigated by passing different volumes of actual water samples as well as ultra-pure water spiked with 1000 μg/L of each compound through the SDB-RPS autotrace-SPE disks while keeping the volume of methanol LC-MS CHROMASOLV^®^ grade (5 mL), the volume of ultra-pure water (5 mL), and the flow rate (1 mL/min) constant [48]. The volumes of spiked ultra-pure water were in the range of 10 mL to 200 mL. The compounds retained by the SDB-RPS autotrace-SPE disks were eluted with methanol (5 mL) before evaporating to dryness using nitrogen gas before reconstituting with methanol (1 mL). For quality assurance, the percentage recoveries of the SPE extracted samples were calculated by comparing the recovered levels with the standard dose levels expressed as a percentage.

### 2.4. Determination of Levels and Occurrence of BPA, CAF, CBZ, HHCB, NP, and AHTN in Water Samples

The SPE extracts were analysed using the GCxGC-HRTOFMS (LECO Corporation, St. Joseph, MI, USA) equipped with a thermal modulator and a split/splitless injector using liquid nitrogen. A low-polarity phase Rxi-5SilMS column (30 m × 0.25 mm i.d., 0.25 µm film thickness) was used for GC in the first dimension analysis (1D GC). The second dimension analysis (2D GC) was performed on a polar Rxi-17SilMS (1 m × 0.25 mm i.d., 0.25 µm film thickness). Helium was used as the carrier gas at a constant linear velocity of 1.9 mL/min. Using an autosampler (Agilent 7890A Series, Santa Clara, CA, USA), splitless injection mode was used with the vaporised sample moving through the injection port liner. The oven temperature was programmed as follows: 50 °C (held for 1 min) ramped to 210 °C at 10 °C/min (held for 2 min) and then ramped to 250 °C at 15 °C/min and held for 10 min. The injector and interface temperatures were set at 220 °C, with the MS quad temperature set at 150 °C and the MS source at 230 °C. The secondary oven was operated at a temperature 5 °C higher than that of the primary oven and was operated in an iso-ramping mode. The modulation period, the hot-pulse duration, and the cool time between stages were set at 3.0 s, 0.4 s, and 1.1 s, respectively. The transfer line to HRTOFMS detector source was operated at 250 °C. The source temperature was 230 °C with a filament bias voltage of −70 eV. The MS mass range was 45 to 550 atomic mass units (amu), with the data acquisition rate at 200 spectra/s, while the detector voltage was 1750 V. The inlet temperature was 200 °C with a modulator offset temperature of 40 °C, and the purge time was 60 s. The mass spectrometer was operated in the positive ion mode with an ionisation voltage of 70 eV using selected ion monitoring (SIM). Prior to injection, the syringe was cleaned five times with n-hexane and once with the sample. An external standard mixture was measured after each batch of five samples to verify instrument measurement performance. Data were processed and consecutively visualised on 2D and 3D chromatograms using LECO ChromaTOF-HRT^®^ software (LECO Corporation, St. Joseph, MI, USA).

### 2.5. Determination of the Mass Accuracies, Limits of Quantification, Limits of Detection, Linearity, and S/N Ratios for BPA, NP, CAF, HHCB, AHTN, and CBZ

Linear regression analysis was used to determine LOD and LOQ for BPA, NP, CAF, HHCB, AHTN, and CBZ based on GC × GC-HRTOFMS’ linear calibration curves for each analyte. It was assumed from the obtained linear calibration curves for each analyte that the GC × GC-HRTOFMS response matrix Y was linearly related to the descriptor matrix X for a limited range of concentrations. The limit of quantification (LOQ) and limit of detection (LOD) for each analyte were thus determined based on the signal-to-noise (S/N) ratios of 10 and 3 based on the residual standard deviation of the response or the standard deviation (SD) of the y-intercept of the regression line of the calibration curve and the sensitivity or slope of the regression line, as shown in Equations (1) and (2):

(1) $$LOD=3.3\;\left(\frac{SD}{Slope}\right)$$

(2) $$LOQ=10\;\left(\frac{SD}{Slope}\right)$$

The LOQ and LOD and tests were performed in triplicate to confirm the accuracy regarding each of the detected EMPs at varying concentrations. The mass accuracies for BPA, NP, CAF, HHCB, AHTN, and CBZ were also obtained directly from GC × GC-HRTOFMS analyses.

### 2.6. Determination of the Temporal and Spatial Variations in the Occurrence of the Analytes during the Study Period

The R statistical software was used to compute descriptive statistics correlation studies, Kruskal Wallis test, ANOVA, and principal component analysis (PCA) utilizing data obtained from both onsite and GC × GC-HRTOFMS analyses of all the 44 water samples over the studied period. Kruskal Wallis test and analysis of variance (ANOVA) were performed to determine temporal variations in the occurrence of analytes in the water samples at 95% confidence level. The PCA and correlation studies were used as quantitative and independent approaches for water classification, allowing the grouping of the water samples and the establishment of correlations between chemical parameters and water samples, respectively. The principal components (PCs) of the PCA were extracted using the Varimax rotation. The total number of PCs to retain was based on the Kaiser criterion, wherein only PCs with eigenvalues >1 were retained and the parameters were retained if their *p*-value < 0.05 at 95% confidence level. Equation (3) shows the R-mode PCA model that was used to compute factor scores:

(3) $$Xj=\overset{p}{\underset{r=1}{\sum }}a_{jr}\;f_{r}\;{Ɛ}_{j}$$

where *f_r_* were the *r*th common factors, *p* was the specified number of factors, *j* was the random variation unique to the original hydrochemical variable *X_j_*, and *a_jr_* was the loading of the *j*th variate on the *r*th factor. The PCA model corresponded to the loading or weights on the extracted PCs. The new factor was expressed as shown using Equation (4)

(4) $$Factor=\;\sum^{}\left(a_{i}\;x\;I_{1}\right)\;$$

where *a_i_* was the loading of *i* index; *I_i_* was the standardized data of *I* index.

The factor score loadings for each water sample were utilised to model spatial variations in the occurrence of the EMPs using Surfer Golden Graphics software for surface mapping (version 8). Specifically, the value of each factor score represented the importance of a given factor at the sampled site. A factor score >+1 reflected sampling areas significantly influenced of EMPs highly loaded in a particular PC. Factor scores <−1 reflected sampling areas virtually unaffected by EMPS highly loaded in a particular PC, whereas near-zero scores reflected areas moderately influenced by EMPs highly loaded in a particular PC. The spatial variations of the occurrence of EMPs highly loaded in a particular PC were assessed by surface mapping contour plots of the factor scores representing each factor.

## 3. Results

### 3.1. Solid Phase Extraction Optimisation Results

#### 3.1.1. Initial Analyte Dose

Autotrace-SPE of actual water samples as well as ultra-pure water samples spiked with initial analyte concentrations of 5 µg/L, 10 µg/L, and 15 µg/L, in triplicate, were investigated. From the results (Table 2), it was observed that the mean percent recovery (*n* = 3) of all the analytes was higher for the samples with an analyte concentration of 10 µg/L than that of samples containing the initial analyte concentration of 5 µg/L. Although the mean percent recovery of analytes for a 15 µg/L initial dose was higher than that of the 5 µg/L initial dose, they were actually found to be lower than those for the 10 µg/L initial dose, possibly due to interferences from other sample matrices, especially in actual water samples. It was thus observed that the 10 µg/L initial dose was the optimal initial dose for autotrace-SPE of all the analytes.

#### 3.1.2. Sample pH

**Figure 2:**
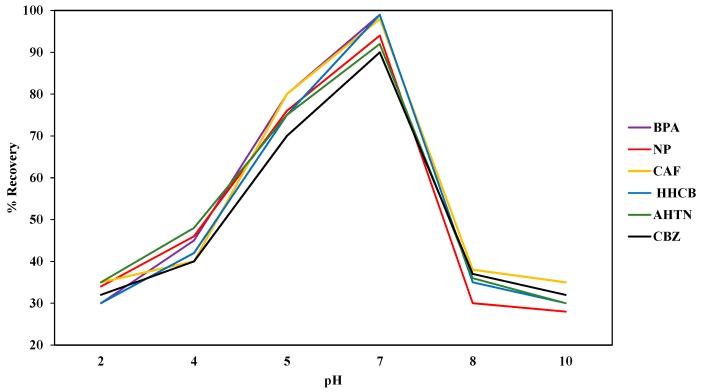
Effect of pH on the mean percent autotrace SPE recovery (*n* = 3) of BPA, NP, CAF, HHCB, AHTN, and CBZ (*n* = 3).

The highest mean percent autotrace-SPE recoveries for the analytes, measured in triplicate, were recorded at pH 7 (Figure 2). Therefore, the optimum pH for the autotrace-SPE recoveries of the analytes was selected as pH 7, the neutral pH. These results are in line with those reported by Santos et al. [48] who obtained a mean recovery of 80% for ketoprofen at neutral pH. On the other hand, Madikizela et al. [47] observed that a low pH is required for the analysis of acidic pharmaceuticals to prevent the dissociation of acidic compounds. However, Madikizela et al. [47] also stated that the sample pH during SPE must not be too low because acidic compounds that interfere in wastewater treatment processes may also be co-extracted and could interfere in the analysis if the sample pH is too low.

#### 3.1.3. Sample Volume

**Figure 3:**
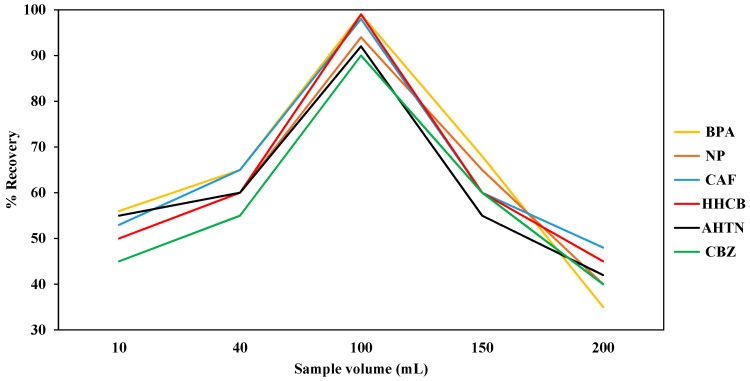
Effect of sample volume of spiked ultra-pure water onto Oasis^®^ HLB cartridge on the mean percent autotrace SPE recoveries of NP, CAF, HHCB, AHTN, and CBZ (*n* = 3).

In this study it was observed that the mean percent autotrace SPE recoveries of all analytes were affected by the volume of the actual water samples as well as ultra-pure water samples loaded into the SDB-RPS autotrace-SPE disks. Specifically, an improvement in the mean percent autotrace-SPE recoveries of each of the analytes was observed when increasing the loading from 10 mL to 100 mL and then a decline was observed when increasing the loading from 100 mL to 200 mL (Figure 3). Therefore, a sample volume of 100 mL was selected as the optimum sample volume for the highest mean percent autotrace-SPE recoveries of the analytes. This is in line with the findings of Madikizela et al. [47] who observed that this trend is as a result of the capacity of the sorbent being exceeded and that higher volumes tend to overload the SPE cartridge and target compounds end up competing for the adsorbent material with matrix interferences. Madikizela et al. [47] further noted that high sample volume may result in the saturation of the SPE sorbent thereby leading to poor percent recoveries.

### 3.2. Mass Accuracies, Limits of Quantification, Limits of Detection, Linearity, and S/N Ratios for BPA, NP, CAF, HHCB, AHTN, and CBZ

For all analytes, good linearity and reproducibility of analyses (R^2^ > 0.99) was achieved (Table 3). All the analytes registered desirable S/N ratios that were much higher than 100:1 (Table 3). The computed LOD, which according to Glaser et al. [49] corresponds to the lowest concentration that can be reliably detected and readily distinguished from zero with a certain degree of confidence, ranged from 0.25 ng/L for HHCB to 1.1 ng/L for CBZ (Table 3). On the other hand, the computed LOQ, which is the lowest amount of analyte that can be quantitatively determined at a definite level of accuracy and/or precision, ranged from 1.2 ng/L for HHCB to 3.9 ng/L for CAF (Table 3). All the analytes registered mass accuracies ranging from −0.97 to 0.52 (Table 3), which were below 1 ppm, the maximum allowable exceptional mass accuracy for GC × GC-HRTOFMS [36].

### 3.3. The GCxGC-HRTOFMS Mass Spectra and Chromatograms, Levels, and Occurence of BPA, NP, CAF, HHCB, AHTN, and CBZ

#### 3.3.1. The GC × GC-HRTOFMS Mass Spectra and Chromatograms of BPA, NP, CAF, HHCB, AHTN, and CBZ

The identification criteria for the analytes (BPA, NP, CAF, HHCB, AHTN, and CBZ) were two-fold in that both a first dimension retention time deviation (±2 s) and a second dimension retention time deviation (±0.5 s) were utilized, with the second taking into account the mass spectra with a similarity factor higher than 600 based on the library search. The similarity factor describes how well the library hit matches the peak when using all small molecule mass spectra in the U.S. National Institute of Standards and Technology (NIST) mass spectral libraries [40]. In the NIST mass spectra search algorithm the similarity is computed using numerical functions [40]. Confirmation of target analytes (BPA, NP, CAF, HHCB, AHTN, and CBZ) was based on the retention time, the accurate mass measurement of the molecular ion, the isotopic pattern, and by automated mass spectral library searches with GC × GC-HRTOFMS (Table 4).

**Figure 4:**
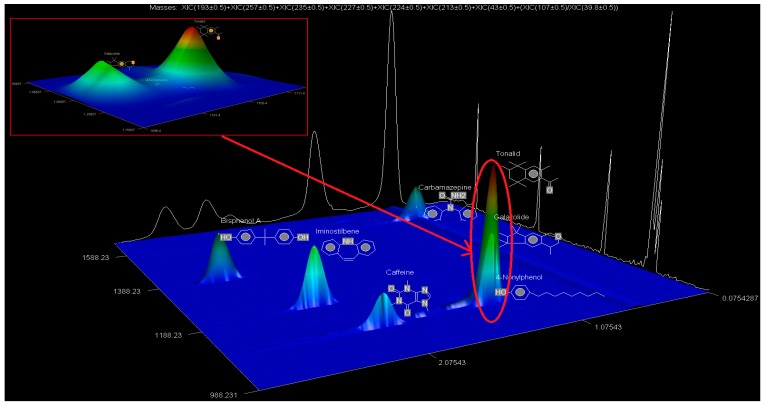
HRTOFMS surface plots of BPA, NP, CAF, CBZ, HHCB, and AHTN spectra obtained from GC × GC-HRTOFMS (two-dimensional gas chromatography coupled to high resolution time-of-flight mass spectrometry) with modulation.

**Figure 5:**
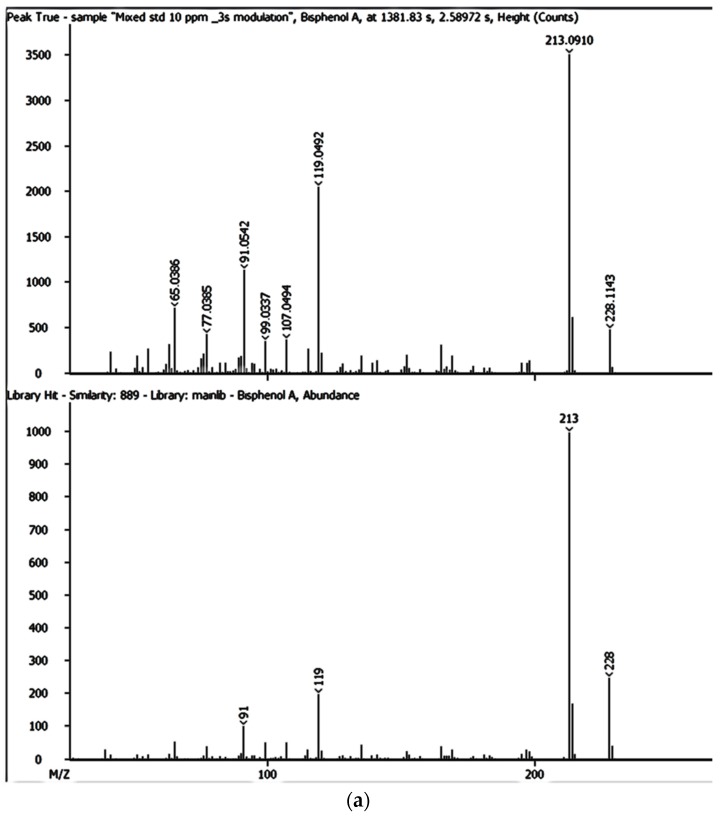

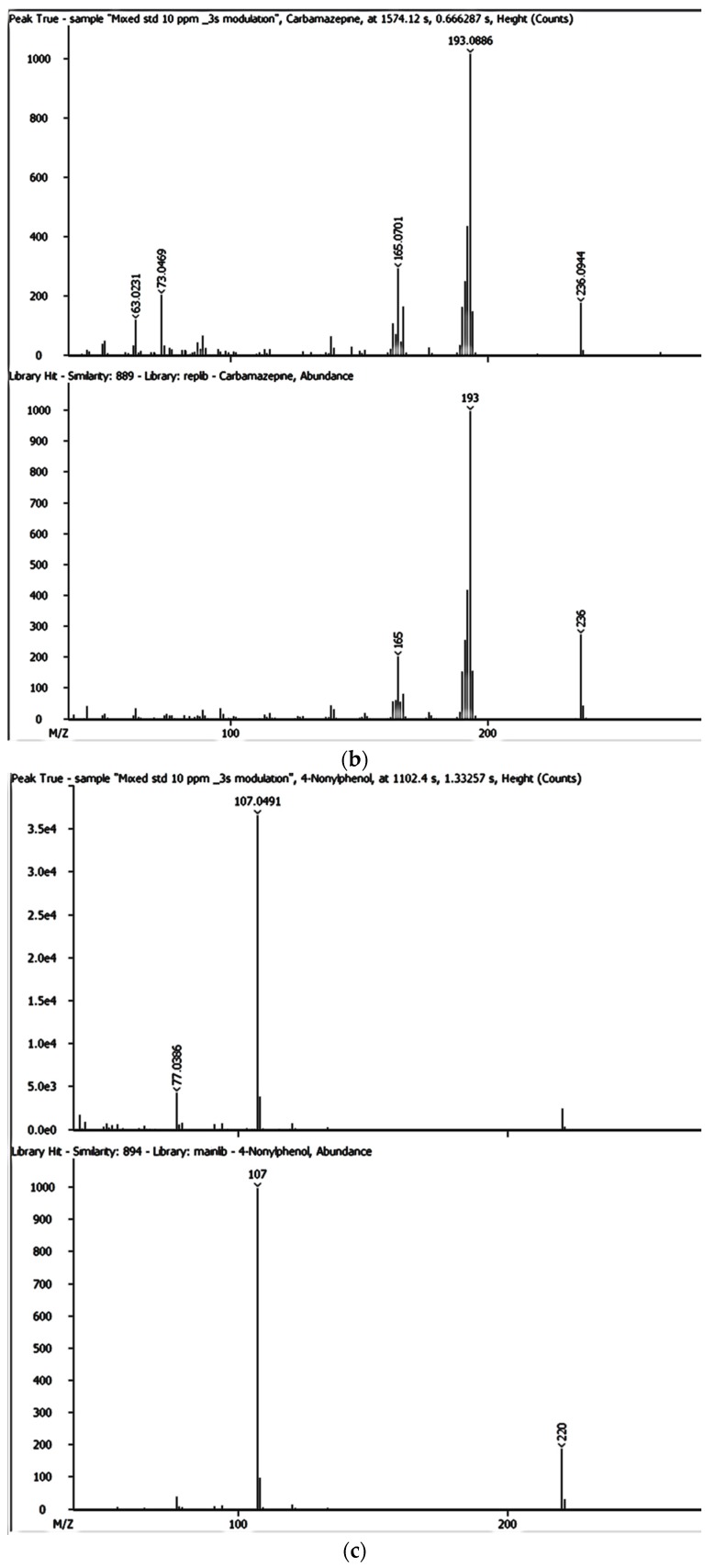

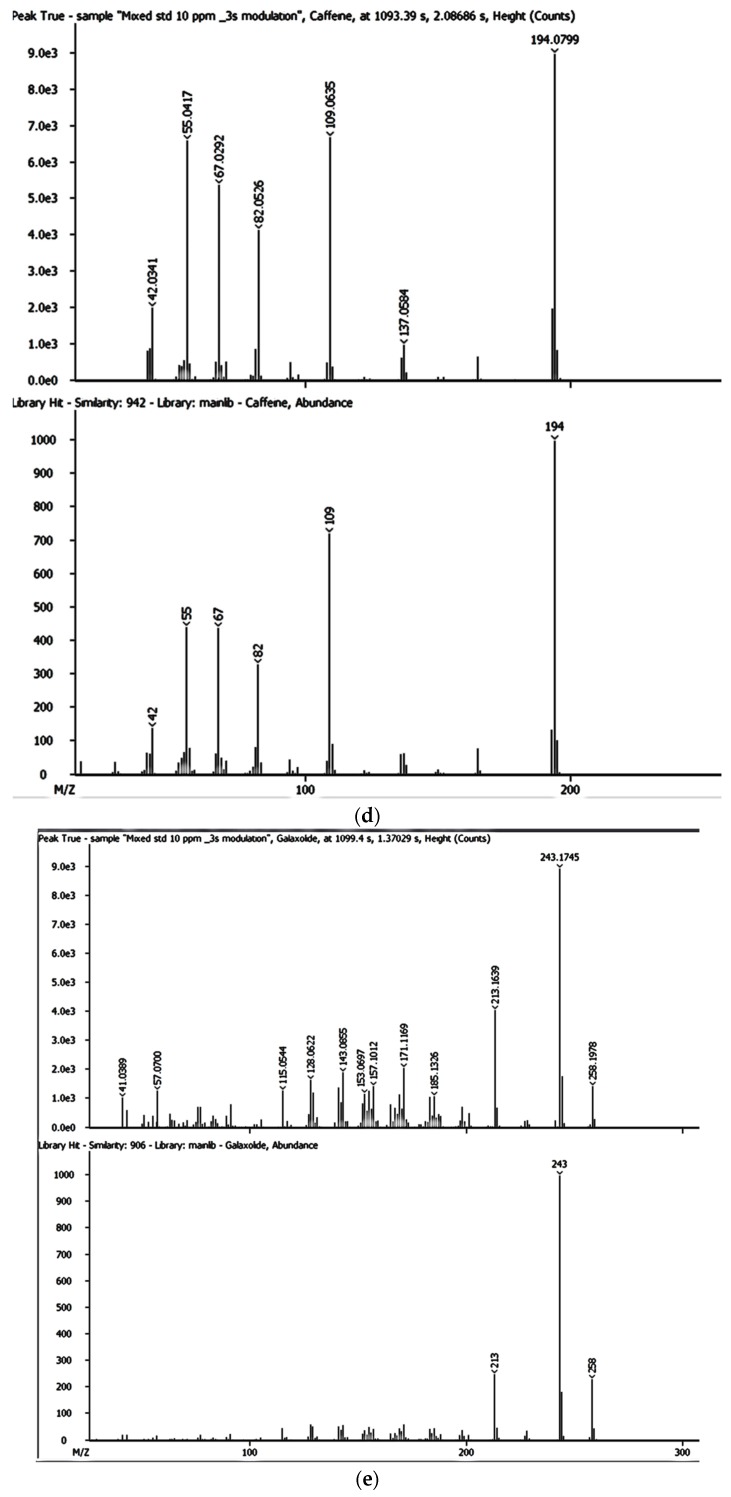

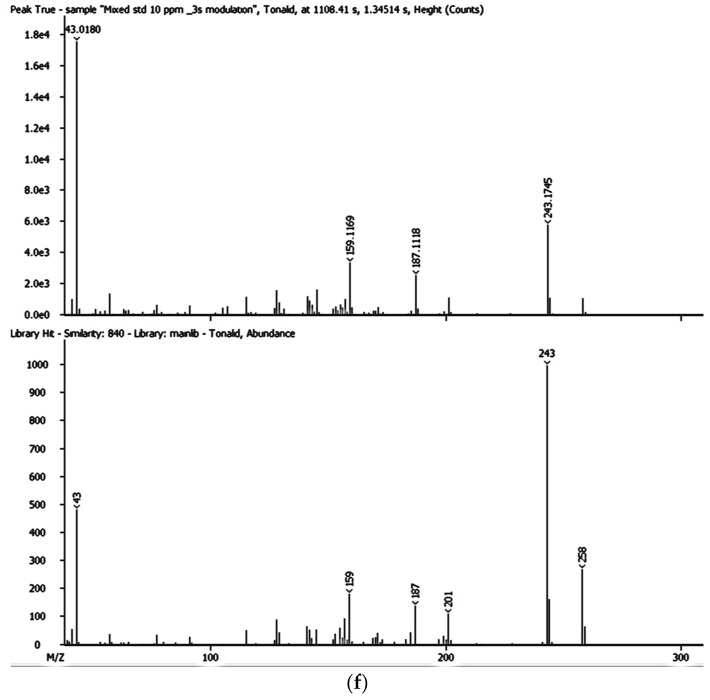
HRTOFMS mass spectra of BPA (**a**); CBZ (**b**); NP (**c**); CAF (**d**); HHCB (**e**); and AHTN (**f**) obtained from GC × GC-HRTOFMS with modulation.

In this study, all the analytes (BPA, NP, CAF, HHCB, AHTN, and CBZ) registered first dimension retention time deviations of ±2 s, second dimension retention time deviations of ±0.5 s, and mass spectra with similarity factors greater than 850, which were much higher than the set 600 identification criterion (Table 4). To this effect all the analytes (BPA, NP, CAF, HHCB, AHTN, and CBZ) met both GG × GC-HRTOFMS identification criteria, and their mass spectra surface plots are presented in Figure 4. In addition, the mass spectra of all analytes obtained from GC × GC-HRTOFMS are presented in Figure 5a–f. The ChromaTOF-HRT^®^ software allowed peak deconvolution of each co-eluting peak, defined a unique ion m/z ratio, and extracted the pure mass spectra of individual analytes across the unresolved area (Figure 4 and Figure 5a–f). Caffeine was observed to be the most polar and volatile EMP of all the analytes and CBZ was the least. The m/z fragments for each analyte are presented in Table 4.

**Figure 6:**
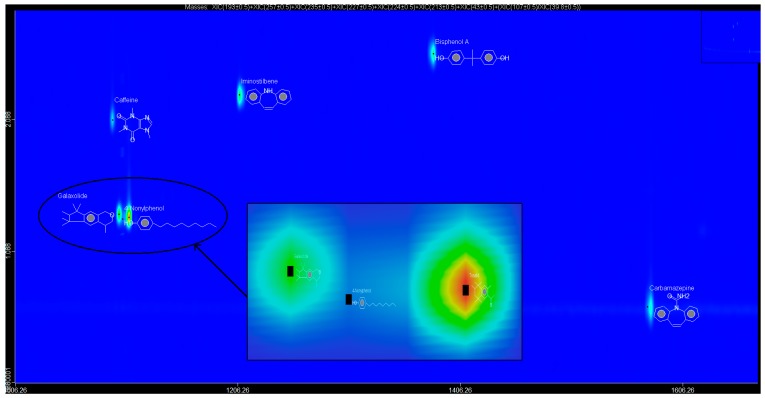
Contour plot of a GC × GC chromatogram of BPA, NP, CAF, AHTN, HHCB and CBZ with the retention times on column 1 (indicating volatility) and column 2 (indicating polarity) on the x- and y-axes, respectively.

The obtained GC × GC-HRTOFMS chromatograms for the analytes BPA, NP, CAF, AHTN, HHCB, and CBZ are presented in the contour plot 2D version (Figure 6). Each spot or peak on the two images represents an individual compound for which a full mass spectrum was available. The retention times in the 1D GC (GC × GC-HRTOFMS without modulation) and 2D GC (GC × GC-HRTOFMS with modulation) and their coordinates in the contour plot were used to identify peaks, or spots in the contour plot. The contour plot of the GC × GC-HRTOFMS chromatogram typically demonstrates the main advantage of a high resolution 2D GC, namely the unique feature of “structured” chromatograms; this structured nature separates compounds into distinct groups for easy identification. It clearly shows the different groups of analytes in certain bands along the 2D plane. The analysis of GC × GC-HRTOFMS contour plot group types is powerful and a major advantage for GC × GC analysis.

Inspection of the 3D surface plot images of the GC × GC-HRTOFMS chromatograms compared with the traditional one-dimensional (1D) version (without modulation) chromatograms indicates that the GC × GC-HRTOFMS has better separation capability and the plot identifies the locations in the 2D separation plane (Figure 6). This also demonstrates the other advantages of GC × GC-HRTOMS, namely “structured” chromatograms, high sensitivity through peak sharpening and creating additional peak capacity as the chromatographic plane is expanded [43,50,51,52,53]. It was further observed that some peaks, such as those of nonylphenol, were invisible in the conventional 1D GC chromatogram (without modulation) but visible in 2D GC (i.e., GCxGC chromatogram with modulation).

#### 3.3.2. Levels and Occurrence of BPA, NP, CAF, HHCB, AHTN, and CBZ in Water

The mean concentrations (measured in triplicate) of BPA, NP, CAF, HHCB, AHTN, and CBZ in water samples from Gauteng, Mpumalanga, and North West provinces have been presented in Table 5. The levels of BPA ranged from a not detectable limit (n.d.) to 181 ± 8.3 ng/L (recorded at Spring 1—Sisukumile Secondary School and Lower Lochiel Community) (Table 5). Elsewhere, studies by Regnery and Püttmann [54], Reinstorf et al. [6], Kim et al. [55], and Peng et al. [56] reported BPA levels in the ranges of 192–215, 192–215, 7.5–334, and 6–881 ng/L, respectively. Generally, the recorded levels of BPA in this study were lower than those reported by Regnery and Püttmann [54], Reinstorf et al. [6], Kim et al. [55], and Peng et al. [56]. The observed occurrence of BPA in the majority of the water sources in this study was attributed to the influence of municipal wastewater. The results are similar to those reported by Furhacker et al. [57]; Fromme et al. [58], and Musolff et al. [8] who also observed that the potential sources of BPA in water are municipal wastewater as well as industrial wastewater. The Minnesota Department of Health (MDH) [59] developed a guidance value of 20 µg/L for BPA. To this effect, a person drinking water with a BPA concentration at or below 20 µg/L would have little or no risk of any health effects from BPA. However, BPA has been reported to be one of the endocrine disrupting compounds with estrogenic receptors in human and animal life even at lower concentrations [8] (Table 1). In this study, all samples were found to have mean BPA concentrations below the lowest guidance value of 20 µg/L BPA [59]. Although the levels of BPA were generally low, the presence of increased BPA levels in some water samples such as the spring at Sisukumile School in the Lochiel Community is a major concern since it is the major source of drinking water, and water used for other domestic purposes (i.e., cooking and washing) for the Sisukumile Secondary School and Lower Lochiel Community. The spring is centrally located and therefore the spring water is most often used by the Lochiel community; however, a lot of litter in the form of plastics, food can liners, and paper was observed in and around the spring. The school (Sisukumile) is located near the spring and there are some agricultural activities and outside pit latrines in the area.

The mean CAF concentrations ranged from n.d. to 82.41 ± 5.1 ng/L (Table 5), with the highest mean concentration (i.e., 82.41 ± 5.1 ng/L) registered in the Mkomazane River, into which the effluent from the Eerstehoek (Elukwatini) WWTP is discharged. Elsewhere, studies by Ternes et al. [60], Musolff et al. [8], and Spongberg et al. [61] reported CAF concentrations in the range of 190 ± 90, 48, and 241, 121, 446 ng/L, respectively. The maximum levels of CAF recorded in this study were generally lower than those reported by Ternes et al. [60] and Spongberg et al. [61]. Nevertheless, the levels of CAF observed in this study could be attributed to an additional constant source of untreated wastewater [60]. In terms of ecotoxicological effects, studies have shown that CAF leads to insomnia (sleep disruption) in humans and other animals, even at trace concentrations [8] (Table 1). The levels of CBZ were highest (i.e., 58 ± 0.2 ng/L) in Eerstehoek WWTP effluent (Table 5). Elsewhere, studies by Regnery and Püttmann [54] and Reinstorf et al. [6] reported CBZ levels in the range of 102–1194 ng/L. The highest levels of CBZ reported by Regnery and Püttmann [54] and Reinstorf et al. [6] were generally higher than those observed in this study. Studies have shown that CBZ has the virtue of disrupting the production of red blood cells, white blood cells, and platelets in humans and other animals, even at lower concentrations [8].

The mean levels of NP ranged from n.d to 98 ± 1.4 ng/L observed in the Eerstehoek WWTP effluent (Table 5). A study by Peng et al. [56] reported NP levels in the range of 36–33,231 ng/L. The recorded levels of NP in this study were generally lower than the maximum levels of NP as reported by Peng et al. [56]. It is worth noting that NP is a constituent of detergents and an anti-oxidant, and it was therefore considered that municipal wastewater would be its main source in water. According to Musolff et al. [8], NP has an endocrine disrupting effect in humans and other animals, even in trace concentrations. The mean HHCB levels were highest in Spring 1—Sisukumile Secondary School, Lower Lochiel Community with the mean concentration ranging from n.d. to 3477 ± 35 ng/L (Table 5). The mean levels AHTN levels were highest (i.e., 98.1 ± 7.11 ng/L) in the Krokodil River at Hartbeespoort 1 (Table 5), although high levels of AHTN were also observed in the effluent, Eerstehoek WWTP. Elsewhere, studies by Regnery and Püttmann [54] and Reinstorf et al. [6] reported HHCB and AHTN levels in the ranges of 35–1814 ng/L and 5–273 ng/L, respectively. The maximum levels of AHTN and HHCB recorded in this study were generally lower than those observed by Regnery and Püttmann [54] and Reinstorf et al. [6]. According to Standley et al. [62], a combination of musk fragrances such as AHTN and HHCB with CAF is a unique tracer for the impact of wastewater and wastewater treatment plant effluents on water resource. Both AHTN and HHCB are both polycyclic musks commonly used in fragrances, and they are known to have a chemo-sensitisation effect, a phenomenon characterised by the inhibition of proper functioning of particular cellular glycoprotein and the production of tumour cells in humans and other animals [8].

To the best of our knowledge, there are no reported guidelines for CAF, NP, CBZ, HHCB, and AHTN for water and wastewater. However, research has shown that among the EMPs, CBZ, HHCB, and AHTN are very resistant to biodegradation [7]. In particular, Schirmer and Schirmer [7] reported that only around 30% of CBZ were bio-transformed when sewage sludge was used as a source of microorganisms for biodegradation of CBZ in long-term (two-year) batch experiments done by UFZ-Department of Analytical Chemistry. It is thus clear that plant and animal life, as well as some communities (especially those living in close proximity to contaminated water sources) are at risk of the effects of the identified EMPs since all the EMPs were present in all types of water samples even though found to be at different concentrations.

### 3.4. Temporal and Spatial Variations in the Levels and Occurrence of BPA, NP, CAF, HHCB, AHTN, and CBZ in Water Samples

#### 3.4.1. Temporal Variations in the Levels and Occurrence of BPA, NP, CAF, HHCB, AHTN, and CBZ in Water Samples

Except for pH (*p*-value = 0.000), the results of the Kruskal-Wallis test and ANOVA revealed that there was no statistically significant temporal variation in the occurrence of BPA, NP, CAF, HHCB, AHTN, and CBZ (*p*-value > 0.05) at the 95% confidence level (Table 6), despite an overall increase in the mean bi-monthly concentrations of all analytes in all water samples between June 2014 and April 2016. It was observed that the levels of pH, TDS, and EC were uniformly and significantly correlated with each other (*p*-value < 0.05), but weakly and statistically insignificantly correlated with the EMPs, BPA, CAF, CBZ, HHCB, AHTN, and NP (*p*-value > 0.05) at the 95% confidence level (Table 7). This suggested that, regardless of time of sample collection, the occurrence and distribution of the EMPs in water collected from Gauteng, Mpumalanga, and North West provinces were largely independent of mineralisation processes in water, as shown by the statistically insignificant correlations with measures of the level of mineralization in water (i.e., TDS and EC). Nevertheless, there was a significant correlation between BPA and HHCB (*r*^2^ = 0.905, *p*-value = 0.000), CAF and NP (*r*^2^ = 0.607, *p*-value = 0.043), CBZ and CAF (*r*^2^ = 0.610, *p*-value = 0.046), and HHCB and AHTN (*r*^2^ = 0.692, *p*-value = 0.014) (Table 7), suggesting that the sources of these EMPs were similar.

#### 3.4.2. Spatial Variations in the Levels and Occurrence of BPA, NP, CAF, HHCB, AHTN and CBZ in Water Samples

Table 8 presents PCs and variable loadings generated by the PCA model. Two PCs, which accounted for 89.99% of the total variance were extracted (Table 8). PC1 explained 51.46% of the variance and accounted for the majority of the variance in the original dataset for levels and occurrence of EMPs in water samples from the studied area. PC2 explained 38.53% the variance in the original dataset for levels and occurrence of EMPs in water samples from the studied area. PC1 registered high positive loadings in CAF, CBZ, and BPA and high negative loadings in pH, TDS, and EC. This suggested that CAF, CBZ, and BPA might have originated from similar sources and that their levels and occurrence in water are weakly affected by mineralization processes in water. This also suggested that the occurrence of CAF, CBZ, and BPA increased with decreases in pH of water. On the other hand, PC2 registered high positive loadings in AHTN, HHCB, and NP. This suggested that AHTN, HHCB, and NP might have originated from similar sources. This is not surprising considering that AHTN, HHCB, and NP are major ingredients of personal care products and detergents.

**Figure 7:**
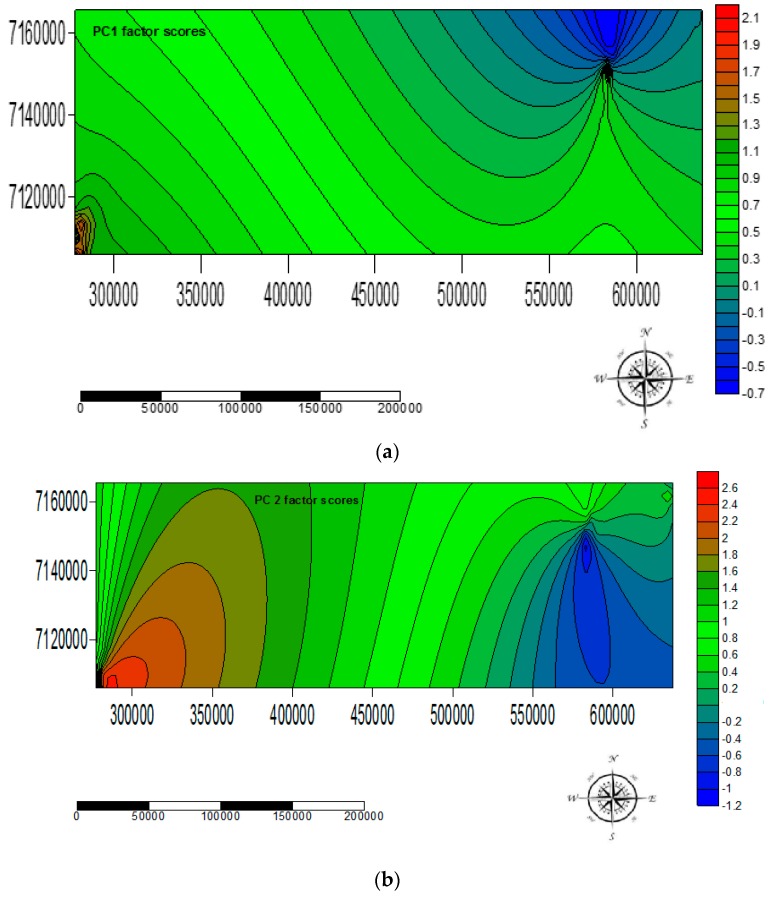
Surface mapping contour plots showing spatial distribution of the PCA analysis factor scores and spatial variations in the occurrence of EMPs with high loadings in (**a**) PC1 and (**b**) PC2.

It was observed that the concentrations of AHTN, CBZ, HHCB, CAF, NP, and BPA in water varied spatially, with BPA being the most widely distributed EMP that was present in 62% of the sampled sites (Table 5). Figure 7 shows the surface mapping contour plots of the PCA factor score model. The surface mapping contour plot showing the spatial distribution of PC1 factor scores has been presented in Figure 7a. Positive PC1 factor scores (i.e., >+1) were observed in water samples collected from Mpumalanga province. The majority of water samples from Gauteng and North West provinces registered negative PC1 factor scores and therefore were unaffected by EMPs highly loaded in PC1. Similarly, the surface mapping contour plot showing the spatial distribution of PC2 factor scores has been presented in Figure 7b. Positive PC2 factor scores (i.e., >+1) were observed in water samples collected from Mpumalanga province. The majority of water samples from Gauteng and North West provinces registered negative PC2 factor scores and therefore were unaffected by EMPs highly loaded in PC2, with a few samples moderately affected by EMPs with high loadings in PC2.

It was clear from the surface mapping contour plots of the PCA factor model that the levels and occurrence of EMPs with water samples collected from Mpumalanga province were higher than the levels of the EMPs registered for their Gauteng and North West counterparts. These higher levels of EMPs in Mpumalanga were attributed to the location, proximity of the sources to sanitary facilities, and open nature of the water sources in Mpumalanga which made them prone to EMP input through litter in the form of plastics, food can liners, and paper in and around the water sources in addition to water sources being prone to wastewater input. Some agricultural activities and outside pit latrines could also have contributed to the higher levels of EMPs in the water sources in Mpumalanga. It was clear that the communities especially in Mpumalanga province were at risk of the effects of the identified EMPs since all the EMPs were present in all types of water samples, though at different concentrations. There is need for better scientific understanding of occurrence, distribution, and environmental fate of EMPs, as well as effective EMP removal technologies in order to protect human health and the environment. These findings agree with those by Reinstorf et al. [6], Musolff et al. [9], Luo et al. [10], Huerta-Fontela et al. [18] and You et al. [63] who reported widespread spatial variations in the occurrence of EMPs, most of which have not been well studied despite the studies on spatial variations and linkages of the same EMPs with existing environmental factors having the virtue of enriching the scientific understanding of behaviour, distribution, and fate of the EMPs in water bodies in addition to assisting direct future management and water pollution safeguards.

## 4. Conclusions

The EMPs, BPA, NP, CAF, HHCB, AHTN, and CBZ were successfully extracted and pre-concentrated using autotrace-SPE prior to determination using the GCxGC-HRTOFMS system in water samples collected from Mpumalanga, Gauteng, and North West provinces, South Africa at better S/N ratios as well as lower LOD and LOQ. Although no statistically significant temporal variation in occurrence of the analytes was observed in water samples at the 95% confidence level, all the analytes were detected, at different levels concentrations, in the different sample types analysed across a broad spectrum of wastewater effluent, surface water, groundwater, and treated water, with BPA found to be present in 62% of the sampling sites and thus identified as the most widely distributed EMP in these water systems. It was also observed that the levels and occurrence of EMPs varied spatially and were a function of two PCs (PC1 and PC2) which controlled 89.99% of the observed variance. The results indicated that the identified EMPs pose ecotoxicological risks to aquatic life as well as communities, especially in Mpumalanga province which was largely influenced by EMPs with high loadings in the two PCs. The results of this study will thus contribute to the body of knowledge on levels and occurrence of EMPs in water, especially in considering the case of Gauteng, North West, and Mpumalanga provinces in South Africa. An understanding of temporal and spatial variations in the levels and occurrence of EMPs in water is critical for enrichment of the scientific understanding of behaviour, distribution, and fate of the EMPs in water bodies necessary for informed decision making on direct future water resources management, water pollution safeguards, as well as regulation of the EMPs in water.

## Figures and Tables

**Table 1:** Some of applications, effects, and properties of Bisphenol A (BPA), 4-Nonylphenol (NP), caffeine (CAF), galaxolide (HHCB), tonalide (AHTN), and carbamazepine (CBZ) in wastewater and drinking water.

Compound	Application	Ecotoxicological Effects	CAS-No	pK_ow_
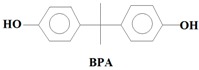	Component in plastics (resins, poly-carbonate), food-can liners, thermal paper (like the kind used for some receipts), and some dental sealants	Endocrine disruptor (estrogenic)	80-05-7	3.32
Formula: C_15_H_16_O_2_				
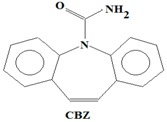	Anti-epileptic drug	Disruption in the production of red blood cells, white blood cells, and platelets	298-46-4	2.45
Formula: C_15_H_12_N_2_O				
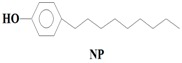	Constituent of detergents, anti-oxidant	Endocrine disruptor, interaction with estrogenic receptor	104-40-5	5.76
Formula: C_15_H_24_O				
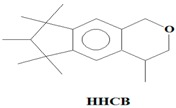	Polycyclic musk (fragrance used in consumer products)	Chemo-sensitizer (interference with transporter proteins (P-glycoprotein) leads to inhibition of the cellular defence mechanism)	1222-05-5	5.9
Formula: C_18_H_26_O				
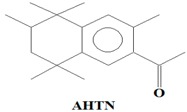	Polycyclic musk (fragrance used in consumer products)	Chemo-sensitizer (interference with transporter proteins (P-glycoprotein) leads to inhibition of the cellular defence mechanism)	1506-02-1	5.7
Formula: C_18_H_26_O				
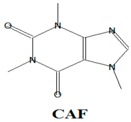	Stimulant, indicator for untreated wastewater inputs	Sleep disruption or insomnia	58-08-2	−0.07
Formula: C_8_H_10_N_4_O_2_				

**Table 2:** The effect of initial dose of analytes on autotrace-SPE recovery of BPA, NP, CAF, HHCB, AHTN, and CBZ (*n* = 3).

Analyte	Initial Dose (5 µg/L)		Initial Dose (10 µg/L)		Initial Dose (15 µg/L)	
	Recovery Levels (µg/L)	Mean Recovery (%)	Recovery Levels (µg/L)	Mean Recovery (%)	Recovery Levels (µg/L)	Mean Recovery (%)
BPA	3.9	78 ± 0.9	9.6	96 ± 2.5	8.2	82 ± 0.82
NP	3.6	72 ± 1.1	9.4	94 ± 1.2	8.5	85 ± 0.56
CAF	3.5	70 ± 2.3	8.2	82 ± 1.5	7.4	74 ± 1.2
HHCB	3.0	60 ± 2.4	8.9	89 ± 1.3	7.1	71 ± 1.8
AHTN	3.1	62 ± 0.5	9.1	91 ± 2.7	8.3	83 ± 0.76
CBZ	3.2	64 ± 2.5	8.3	83 ± 2.1	6.8	68 ± 1.2

**Table 3:** Computed mass accuracies, limits of quantification (LOQ), limits of detection (LOD), linearity, and S/N ratios for BPA, NP, CAF, HHCB, AHTN, and CBZ.

Analyte	S/N Ratio	Mass Accuracy (ppm)	LOQ (ng/L)	LOD (ng/L)	Linearity, R^2^ (*n* = 3)
BPA	4543	−0.97	1.3	0.45	0.999
NP	6497	0.42	2.8	0.94	0.999
CAF	6276	0.52	3.9	1.1	0.999
HHCB	8650	−0.07	1.2	0.25	0.999
AHTN	9459	0.33	1.3	0.39	0.998
CBZ	1758	0.07	3.13	1.0	0.997

**Table 4:** Retention times, similarity, and m/z fragments for BPA, NP, CAF, HHCB, AHTN, and CBZ.

Analyte	Mass	Unique Mass *	Retention Time (s)	Similarity	*m*/*z* or Adduct Ion	Fragment
BPA	228.1143	213.091	1381.83, 2.58972	889	228.1143	Molecular ion (M^+^)
					213.0910	Unique mass
					119.0492	(M^+^ − C_7_H_7_O)-H_2_
					107.0494	(C_7_H_7_O)^+^
					99.0337	(M^+^ − C_9_H_5_O)
					77.0385	(Ar)
					65.0386	(M^+^ − C_10_H_11_O_2_)
CBZ	236.0694	193.089	1574.12, 0.66629	889	236.0944	Molecular ion (M^+^)
					193.0886	Unique mass
					165.0701	(M^+^ − H)-C_2_H_2_N_2_O
					73.0469	(M^+^ + H)-C_8_H_6_N_2_
					63.0231	(M^+^ + H)-C_10_H_8_N_2_O
NP	220.1822	107.049	1102.40, 1.33257	894	220.1822	Molecular ion (M^+^)
					107.0491	Unique mass
					77.0386	(Ar)
CAF	194.0799	109.064	1093.39, 2.08686	942	194.0799	Molecular ion (M^+^)
					109.0635	Unique mass
					137.0584	(M^+^ − CH_2_COH_3_)
					82.0526	(M^+^ − CH_2_CH_2_NHNHO_2_)
					55.0417	CH_2_COCH
					42.0341	–CH_3_CH_2_CH-
HHCB	258.1978	243.175	1099.40, 1.37029	906	258.1978	Molecular ion (M^+^)
					243.1745	Unique mass
					213.1639	(M^+^ − H)-C_3_H_8_
					185.1326	(M^+^ − Ar) + H_2_
					171.1169	(M^+^ − H)-C_6_H_14_
					157.1102	(M^+^ − Ar)-C_2_H_2_
					153.0697	((M^+^ − H)-Ar)-H_2_CH_3_
					128.0622	(Ar-CO-CH_2_-CH-)
					115.0544	(Ar + C_3_H_3_)-H
					57.0700	(CH_3_CHOCH_2_)
					41.0389	-CH_2_CH_2_CH-
AHTN	258.1978	43.0179	1108.41, 1.34514	840	258.1978	Molecular ion (M^+^)
					43.01798	Unique mass
					243.1745	(M^+^ − CH_3_)
					187.1118	(M^+^ − Ar) + 3H^+^
					159.1169	(M^+^ − Ar)-CH_5_O

* quantifier ion.

**Table 5:** Mean concentration (measured in triplicate) of a given emerging micro-pollutant in wastewater and drinking water from Gauteng, Mpumalanga, and North West provinces.

Sampling Site	Province	Mean Concentrations (ng/L) ± SD					
		BPA	CAF	CBZ	HHCB	AHTN	NP
Treated water—Eerstehoek WTP	Mpumalanga	4.0 ± 1	6.4 ± 1.2	n.d.	n.d.	n.d.	n.d.
Raw water—Eerstehoek WTP	Mpumalanga	20 ± 0.2	9.1 ± 0.5	n.d.	n.d.	n.d.	n.d.
Effluent—Eerstehoek WWTP	Mpumalanga	20 ± 4	11 ± 0.5	58 ± 0.2	19 ± 3	96 ± 0.9	98 ± 1.4
Mkomazane River	Mpumalanga	13 ± 0.8	82.41 ± 5.1	29 ± 0.9	9 ± 0.6	17 ± 0.3	12 ± 0.2
Spring 1—Sisukumile Secondary School and Lower Lochiel Community	Mpumalanga	181 ± 8.3	n.d.	n.d.	3477 ± 35	26 ± 1.0	n.d.
Spring 2—Upper Lochiel Community	Mpumalanga	n.d.	n.d.	n.d.	n.d.	n.d.	n.d.
Masakhane Primary School	Mpumalanga	n.d.	n.d.	n.d.	n.d.	n.d.	n.d.
Lipoponyane River	Mpumalanga	9.3 ± 0.6	2.5 ± 0.3	24 ± 0.5	7.2 ± 1.4	13 ± 0.1	n.d.
Sisukumile water tank (JoJo)	Mpumalanga	34 ± 1.8	n.d.	n.d.	n.d.	22 ± 0.7	n.d.
Schoemansville—raw water	North West	0.8 ± 0.2	1.1 ± 0.0	2.3 ± 0.1	n.d.	n.d.	n.d.
Schoemansville—polymer dosing point	North West	n.d.	n.d.	n.d.	n.d.	n.d.	n.d.
Schoemansville—after flocculation	North West	n.d.	n.d.	n.d.	n.d.	n.d.	n.d.
Schoemansville—treated water	North West	n.d.	n.d.	n.d.	n.d.	n.d.	n.d.
Roodeplat dam 10	Gauteng	5.1 ± 0.5	n.d.	1.75 ± 0.3	n.d.	7.77 ± 2.2	4.27 ± 0.5
Roodeplat dam 15	Gauteng	5.0 ± 0.8	n.d.	49.4 ± 8.6	0.3 ± 0.01	7.24 ± 3.2	1.35 ± 0.02
Roodeplat dam 20	Gauteng	5.2 ± 0.8	n.d.	24.27 ± 5.2	1.03 ± 0.3	n.d.	2.23 ± 0.6
Roodeplat dam 25	Gauteng	5.8 ± 1	2.23 ± 0.2	46.87 ± 9.3	59.74 ± 8.7	11.18 ± 3.2	5.38 ± 0.8
Roodeplat dam 30	Gauteng	6.1 ± 0.2	11.31 ± 5.1	10.83 ± 2.2	17.93 ± 4.9	19.73 ± 1.3	28.72 ± 5.2
Roodeplat dam 35	Gauteng	6.4 ± 0.3	4.72 ± 1.0	n.d.	18.26 ± 2.9	56.64 ± 8.8	24.82 ± 9.7
Roodeplat dam 40	Gauteng	4.1 ± 0.5	8.72 ± 0.56	35.25 ± 6.4	6.49 ± 1.8	14.02 ± 3.2	2.87 ± 0.9
Roodeplat dam 50	Gauteng	3.0 ± 0.8	10.6 ± 1.7	23.69 ± 4.5	36.2 ± 8.3	72.71 ± 10.1	n.d.
Roodeplat dam 55	Gauteng	5.2 ± 0.8	1.55 ± 0.6	37.45 ± 7.1	37.77 ± 5.6	82.69 ± 7.3	5.47 ± 1.4
Roodeplat dam 60	Gauteng	6.8 ± 1.2	n.d.	11.04 ± 1.2	46.88 ± 9.0	20.92 ± 7.5	19.56 ± 2.5
Roodeplat dam 65	Gauteng	n.d.	n.d.	9.66 ± 2.4	65.59 ± 4.8	57.09 ± 8.2	5.13 ± 0.9
Roodeplat dam 70	Gauteng	n.d.	n.d.	38.08 ± 3.1	100.94 ± 10.4	3.09 ± 0.75	n.d.
Roodeplat dam 75	Gauteng	n.d.	n.d.	6.78 ± 0.81	26.97 ± 4.57	5.62 ± 1.4	8.33 ± 13.2
Roodeplat dam 80	Gauteng	n.d.	n.d.	1.6 ± 0.02	2.37 ± 1.2	64.75 ± 6.78	1.22 ± 0.06
Roodeplat dam 85	Gauteng	30.34 ± 8.3	5.84 ± 3.7	7.7 ± 0.08	3.02 ± 1.1	27.59 ± 5.7	n.d.
Hartbeespoort 1-Krokodil River	North West	81.24 ± 3.2	n.d.	8.58 ± 2.8	18.08 ± 1.9	98.1 ± 7.11	n.d.
Hartbeespoort 2-HBD Inlet-Krokodil River	North West	36.67 ± 7.3	5.14 ± 1.1	6.76 ± 3.8	9.83 ± 3.2	63.58 ± 0.5	2.57 ± 0.5
Hartbeespoort 3-Hartbeerspoort dam Wall	North West	12.31 ± 5.7	n.d.	1.74 ± 0.66	69.52 ± 14.8	10.95 ± 3.23	n.d.
Hartbeespoort 4-HBD/Krokodil River	North West	5.11 ± 1.22	n.d.	13.99 ± 5.1	n.d.	85.41 ± 9.76	n.d.
Hartbeespoort 5-Snake Park	North West	n.d.	n.d.	1.28 ± 0.4	129.37 ± 15.9	3.65 ± 1.1	n.d.
Hartbeespoort 6-HBD/Krokodil/Megalies	North West	7.34 ± 2.1	n.d.	n.d.	0.45 ± 0.005	75.84 ± 7.93	n.d.
Hartbeespoort 7	North West	n.d.	2.49 ± 0.6	25.71 ± 6.32	2.86 ± 1.32	54.46 ± 9.7	n.d.
Hartbeespoort 8-Megalies River	North West	3.15 ± 2.3	24.52 ± 2.6	52.35 ± 8.6	n.d.	27.44 ± 7.4	3.43 ± 1.3
Hartbeespoort 9-HBD inlet-Megalies River	North West	1.52 ± 0.8	1.43 ± 0.9	30.52 ± 11.5	13.74 ± 5.34	27.18 ± 6.35	n.d.
Hartbeespoort 11-Harties Boat Park	North West	n.d.	n.d.	1.3 ± 0.32	29.91 ± 7.72	46.85 ± 7.8	n.d.
Hartbeespoort 12-Mangwanani	North West	n.d.	n.d.	9.22 ± 2.53	82.43 ± 5.13	13.96 ± 3.89	n.d.
Hartbeespoort 14	North West	n.d.	5.73 ± 0.7	18.79 ± 5.71	n.d.	69.82 ± 8.8	1.35 ± 0.05
Hartbeespoort 15	North West	n.d.	n.d.	n.d.	n.d.	75.84 ± 18.4	n.d.

**Table 6:** Descriptive statistics showing the *p*-value (at 95% confidence level), minimum, maximum, and mean concentration of a given emerging micro-pollutant in wastewater and drinking water from Gauteng, Mpumalanga, and North West provinces over the study period.

Analyte	Minimum	Maximum	Mean	*p*-Value
pH	6.00	9.20	7.48 ± 0.82	0.113
EC (µS/cm)	20.43	993.00	310.49 ± 285	0.000
TDS (mg/L)	3.00	569.00	130.01 ± 112	0.065
BPA (ng/L)	n.d.	181.00	28.55 ± 44.7	0.686
CAF (ng/L)	n.d.	82.41	11.22 ± 18.8	0.638
CBZ (ng/L)	n.d.	58.35	19.63 ± 16.8	0.187
HHCB (ng/L)	n.d.	3477.00	158.49 ± 662	0.057
AHTN (ng/L)	n.d.	98.10	40.44 ± 28.9	0.199
NP (ng/L)	n.d.	98.33	13.75 ± 23.6	0.669

**Table 7:** Correlation studies of the emerging micropollutants and selected physicochemical water quality parameters.

Parameter/*r*^2^/Sig.		pH	EC	TDS	BPA	CAF	CBZ	HHCB	AHTN	NP
pH	*r*^2^	1	0.605 **	0.540 **	0.037	0.007	0.001	0.071	−0.076	0.223
	Sig.		0.000	0.004	0.892	0.979	0.995	0.725	0.681	0.0390
EC	*r*^2^	0.605 **	1	0.574 **	−0.082	−0.222	−0.036	0.170	0.085	−0.167
	Sig.	0.000		0.002	0.764	0.375	0.853	0.396	0.645	0.523
TDS	*r*^2^	0.540 **	0.574 **	1	−0.145	−0.029	−0.201	−0.047	0.093	−0.278
	Sig.	0.004	0.002		0.591	0.909	0.296	0.815	0.614	0.280
BPA	*r*^2^	0.037	−0.082	−0.145	1	−0.048	−0.321	0.905 **	−0.088	−0.110
	Sig.	0.892	0.764	0.591		0.896	0.336	0.000	0.775	0.890
CAF	*r*^2^	0.007	−0.222	−0.029	−0.048	1	0.610	−0.050	−0.237	0.607 **
	Sig.	0.979	0.375	0.909	0.896		0.046	0.872	0.395	0.043
CBZ	*r*^2^	0.001	−0.036	−0.201	−0.321	0.610	1	−0.158	−0.103	−0.265
	Sig.	0.995	0.853	0.296	0.336	0.046		0.462	0.609	0.321
HHCB	*r*^2^	0.071	0.170	−0.047	0.905 **	−0.050	−0.158	1	0.692 **	0.101
	Sig.	0.725	0.396	0.815	0.000	0.872	0.462		0.014	0.731
AHTN	*r*^2^	−0.076	0.085	0.093	−0.088	−0.237	−0.103	0.692 **	1	−0.444
	Sig.	0.681	0.645	0.614	0.775	0.395	0.609	0.014		0.085
NP	*r*^2^	0.223	−0.167	−0.278	−0.110	0.607 **	−0.265	0.101	−0.444	1
	Sig.	0.390	0.523	0.280	0.890	0.043	0.321	0.731	0.085	

** Pearson correlation (*r*^2^) is significant at the 95% confidence level (two-tailed).

**Table 8:** Principal components and variable loadings extracted using the principal component analysis using Varimax rotation.

Variable	Principal Component 1 Loadings	Principal Component 2 Loadings
pH	−0.998	
Total dissolved solids	−0.992	
Electrical Conductivity	−0.962	
Caffeine	0.980	
Carbamazepine	0.935	
Tonalide		0.997
Galaxolide		0.961
Bisphenol A	0.906	
Nonylphenol		0.902
**% Variance**	**51.46**	**38.53**
**Cumulative %**	**51.46**	**89.99**
